# Research on adaptive impedance control technology of upper limb rehabilitation robot based on impedance parameter prediction

**DOI:** 10.3389/fbioe.2023.1332689

**Published:** 2024-01-03

**Authors:** Yuling Zhang, Tong Li, Haoran Tao, Fengchen Liu, Bingshan Hu, Minghui Wu, Hongliu Yu

**Affiliations:** ^1^ School of Health Science and Engineering, University of Shanghai for Science and Technology, Shanghai, China; ^2^ Shanghai Engineering Research Center of Assistive Devices, Shanghai, China; ^3^ School of Mechanical and Automotive Engineering, Shanghai University of Engineering Science, Shanghai, China

**Keywords:** rehabilitation robot, upper limb, impedance identification, adaptive impedance control, optimal stiffness

## Abstract

**Introduction:** With the aggravation of aging and the growing number of stroke patients suffering from hemiplegia in China, rehabilitation robots have become an integral part of rehabilitation training. However, traditional rehabilitation robots cannot modify the training parameters adaptively to match the upper limbs’ rehabilitation status automatically and apply them in rehabilitation training effectively, which will improve the efficacy of rehabilitation training.

**Methods:** In this study, a two-degree-of-freedom flexible drive joint rehabilitation robot platform was built. The forgetting factor recursive least squares method (FFRLS) was utilized to estimate the impedance parameters of human upper limb end. A reward function was established to select the optimal stiffness parameters of the rehabilitation robot.

**Results:** The results confirmed the effectiveness of the adaptive impedance control strategy. The findings of the adaptive impedance control studies showed that the adaptive impedance control had a significantly greater reward than the constant impedance control, which was in line with the simulation results of the variable impedance control. Moreover, it was observed that the levels of robot assistance could be suitably modified based on the subject’s different participation.

**Discussion:** The results facilitated stroke patients’ upper limb rehabilitation by enabling the rehabilitation robot to adaptively change the impedance parameters according to the functional status of the affected limb. In clinic therapy, the proposed control strategy may help to adjust the reward function for different patients to improve the rehabilitation efficacy eventually.

## 1 Introduction

Stroke is globally recognized as the second leading cause of both disability and mortality (Sun et al., 2022). The incidence of stroke worldwide reached 13.7 million new cases, with China alone accounting for 3.94 million new cases (Ma et al., 2021; Vasu et al., 2021). The severity of stroke affects the probability of hemiplegia, as well as the changes in gait speed, balance, spasticity, and range of motion (Hong et al., 2018). With the aggravation of aging and the growing number of stroke patients suffering from hemiplegia in China, the impact of stroke is becoming increasingly noticeable (Honghai et al., 2022). The current number of rehabilitation physicians and therapists is hard to meet the needs of rehabilitation training for the numerous hemiplegic patients. The rehabilitation robot is the outcome of the fusion between robot technology and rehabilitation engineering, which may assist patients with rehabilitation training to a great extent by replacing rehabilitation physicians. Fabio *et al.* proved the feasibility and effectiveness of hand rehabilitation assisted by rehabilitation robot (Vanoglio et al., 2016). Rehabilitation robot offers several advantages over traditional therapy performed by therapists, including consistent delivery of therapy, objective and quantitative assessment, and virtual reality interfaces to enhance the rehabilitation experience (Wang et al., 2019). The traditional upper limb rehabilitation robot can only perform the programmed rehabilitation movements repeatedly, lacking the ability to adaptively adjust the training parameters based on the affected limb’s participation during active rehabilitation training. Therefore, robot-assisted rehabilitation can more effectively motivate patients to complete their rehabilitation training (Islam et al., 2021).

The impedance parameter of the upper limb is a useful method to evaluate the extent of the affected limb’s engagement in rehabilitation exercises, and impedance control is a widely-used technique for regulating the levels of assistance provided by robotic systems during rehabilitation training (Perez-Ibarra et al., 2015). In order to provide appropriate assistant force in training, different control strategies have been proposed by relevant studies. Perez Ibarra *et al.* conducted two adaptive impedance control strategies and indicated that incorporating the damping parameters of patients into the patient impedance model could enhance the velocity correlation (Perez-Ibarra et al., 2019). Krebs *et al.* developed an impedance control algorithm based on performance metrics such as speed, time, or EMG signals to adaptively adjust the duration and levels of assistance provided by the robot during movement (Krebs et al., 2003). In order to adjust the interaction change between the human-machine system, Wolbrecht combined the model-based adaptive impedance control with real-time torque calculation as feed-forward for the affected limb (Wolbrecht et al., 2008). Losey *et al.* proposed a sensorless force estimation component to evaluate the patient’s ability state and subsequently modified the training mode of the rehabilitation platform (Pehlivan et al., 2016). Although the resistance training for stroke patients has become a popular method to facilitate rehabilitation, most rehabilitation robots’ resistance training offers constant resistance, which lacks adaptability to the patients’ variable status.

Some studies considered the adaptation of resistance in robot-assisted rehabilitation. Guozheng Xu used the biological damping and stiffness parameters identified online to monitor the changes of muscle strength of the subjects automatically and modified the required resistance to be aligned with the changes in the muscle strength of the subjects (Xu et al., 2017). OttC proposed a control framework for passive flexible joint rehabilitation robot and designed the impedance controller which was verified on the DLR lightweight robots and was only suitable for the cases of constant impedance parameters (Albu-Schaffer et al., 2007). Researchers from the Chinese University of Hong Kong suggested an iterative learning impedance controller for rehabilitation robots, providing a theoretical basis to ensure dynamic stability in variable impedance control driven by compliance-driven rehabilitation robots (Li et al., 2018). A nonlinear model relating to an adaptive bilateral impedance controller was proposed by Mojtaba Sharifi’s group, which was suitable for various collaborative tele-rehabilitation of patient-rehabilitation physician interaction in a multi-degree of freedom tele-robotics system (Sharifi et al., 2017). Adaptive impedance control also played a role in exoskeleton rehabilitation robots, using a nonlinear time-delay disturbance observer (Brahmi et al., 2021). In the current rehabilitation robotics studies, the existing human impedance parameter identification methods can hardly identify the impedance parameters of human upper limb in real time and apply them in rehabilitation training dynamically and effectively.

In the process of rehabilitation training, more and more people consider the importance of variable impedance for rehabilitation training, and the interaction force between human-machine system to make accurate evaluation of the patient’s state. However, the present training model still cannot mobilize the participation of patients. If the rehabilitation robot can identify the impedance parameters of the upper limb end and modify the rehabilitation strategy by adjusting the impedance parameters of the rehabilitation robot adaptively according to the patient’s status, the rehabilitation efficiency can be improved significantly, which is more conducive to the rehabilitation of the affected limb.

In this study, aiming to increase the effectiveness of upper limb rehabilitation robot, a robot rehabilitation platform was established and an adaptive impedance control strategy was proposed, which could adaptively change the impedance parameters according to the subject’s participation. The paper is organized as follows: Section II describes a mechanical platform of rehabilitation robot built for the following study and the adaptive impedance control strategy. Section III demonstrates the simulation verification and the experiment results. Section IV conducts the discussion about the results, and Section V draws the conclusion of the study.

## 2 Materials and methods

### 2.1 Rehabilitation robot system

#### 2.1.1 Mechanical platform and control system

**FIGURE 1:**
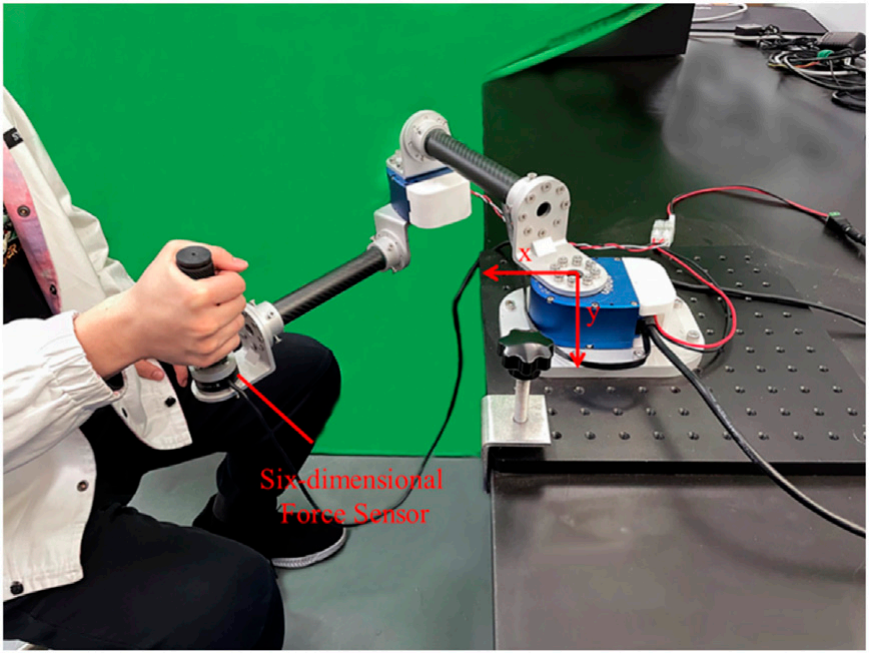
The flexible upper limb rehabilitation robot.

As shown in Figure 1, the flexible joint rehabilitation robot platform was constructed. Based on the two-degree-of-freedom flexible joint upper limb rehabilitation robot, two connecting rods were coupled in series using a flexible driver. Tube A and B were made of carbon fiber tubes, which had the advantages of lightweight and strong material. The end force sensor adopted the SRI’s six-axis (force and moment) force sensor M3714A, which could simultaneously measure the force and moment in the end of Cartesian coordinate system. The robotic joint was one of Seenpin’s XGA series. The joint integrated the motor, reducer, elastomer, controller, and a variety of sensors. The joint was characterized by high power density, high speed, and a high torque output.

The external bus control was applied on the platform. The host and the joint were connected by a network cable. The signal transmission between the two joints and the host was achieved by Ethernet communication. The control system supported MATLAB one-stop development environment, which reduced the time cost of debugging the underlying hardware and network construction for the experiment. Key joint parameters were shown in the following Table 1. The stiffness of the joint adopted in the experiment was 170 Nm/rad.

**TABLE 1:** XGA key joint parameters.

Configuration	XGA
Maximum torque	19Nm
Maximum speed	28.5RPM
weight	550g
Transmission ratio	766.222:1
communication	Ethernet
sensor	Detect torque, acceleration, temperature and current

#### 2.1.2 Robot kinematics model

**FIGURE 2:**
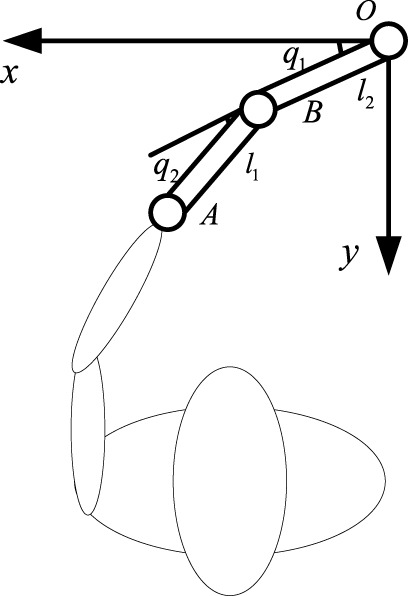
The training diagram of the two-degree-of-freedom flexible joint upper limb rehabilitation robot.

The training diagram of the two-degree-of-freedom flexible joint upper limb rehabilitation robot could be simplified as Figure 2. The upper limb rehabilitation robot was composed of two rods (rod A and rod B), $m_{1}$ = 1 kg, $m_{2}$ = 0.7 kg, $l_{1}=l_{2}=0.4$ , $l_{c1}=l_{c2}=0.2$ . *l*_*c1*_ *and l*_*c2*_ were the centroids of the two rods respectively. *l*_*1*_ and *l*_*2*_ were the lengths of the two rods respectively. Assuming the two rods had the same mass, the midpoints of rods A and B served as the mass centers of the two rods respectively, and *q*_*1*_ *, q*_*2*_ represented the joint angles of rod A and rod B. With point O as the center, the forward kinematics formula of the upper limb rehabilitation robot with two degrees of freedom was established as follows.

$$x_{p}=l_{1}\cos q_{1}+l_{2}\cos\left(q_{1}+q_{2}\right)$$ (1)

$$y_{p}=l_{1}\sin q_{1}+l_{2}\sin\left(q_{1}+q_{2}\right)$$ (2)

$x_{p}$ and $y_{p}$ were the horizontal and vertical coordinates of the Cartesian space of the robot end.

The inverse kinematics formula was derived from the forward kinematics:

$$q_{1}=\mathrm{atan}2\left(-l_{2}s_{2}x_{e}+\left(l_{1}+l_{2}c_{2}\right)y_{e},\left(l_{1}+l_{2}c_{2}\right)x_{e}+l_{2}s_{2}y_{e}\right)$$ (3)

$$q_{2}=\pm \mathrm{acos}\left(\frac{x_{p}^{2}+y_{p}^{2}+l_{1}^{2}-l_{2}^{2}}{2l_{1}l_{2}}\right)$$ (4)

### 2.2 Adaptive impedance control strategy

**FIGURE 3:**
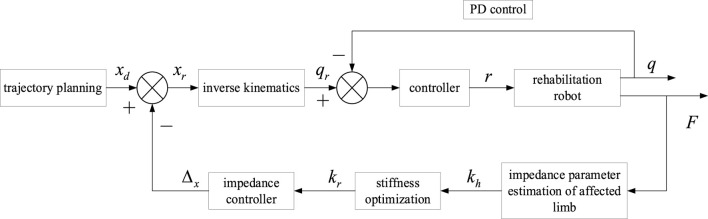
The adaptive impedance control diagram based on human impedance parameter identification.

The adaptive impedance control diagram based on human impedance parameter identification was shown in Figure 3, which mainly included impedance parameter estimation of the affected limb, stiffness optimization, impedance controller, trajectory planning, inverse kinematics, and robot controller, etc. The robot first determined the rehabilitation task, chose the task node, carried out trajectory planning for the rehabilitation robot through quintic polynomial interpolation to get the expected end trajectory *X*_*d*_, and then calculated the joint expected trajectory through inverse kinematics *q*_*d*_ as the controller input. The position of the joint controller was regulated by PD control. Next, the impedance parameters *K*_*h*_ of the affected limb were identified online using the FFRLS. The impedance parameters of the upper limb end were also acquired. The optimal impedance *K*_*r*_ was calculated by equations (14) and (15), and the terminal position correction Δx was obtained by inputting *K*_*r*_ into the impedance controller, correcting the expected trajectory *X*_*d*_ to the reference trajectory *X*_*r*_. The above process was the adaptive impedance control procedure.

#### 2.2.1 Identification of upper limb impedance parameters

Some studies have considered mechanical impedance control as an important method of human motion control. The complex human arm model was simplified as a Cartesian impedance model. The internal model of the arm was transferred to the end of the human arm in the horizontal plane. Therefore, stiffness, damping, and mass became the three components of the mechanical impedance at the end of the human upper limb, relating to force, position, speed, and acceleration respectively. In order to use this model to assume human-computer interaction in the rehabilitation system, it was necessary to estimate the impedance at the end of the human arm. In this section, a model of human upper limb was established and the impedance at the end of human upper limb was estimated by FFRLS.

**FIGURE 4:**
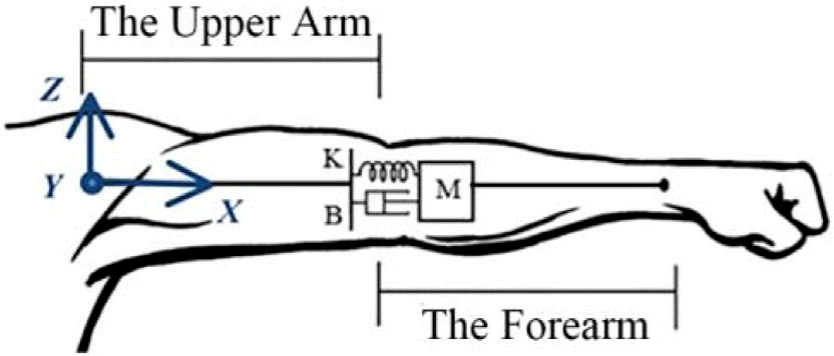
The impedance model of the human upper limb.

Since the musculoskeletal system was assumed to be a mass-spring-damping system, the dynamic motion equation of the mass-spring-damper system was used as a mathematical model to measure the dynamic impedance of the upper limb. The impedance model of the upper limb was depicted in Figure 4, which could be used to measure the dynamic impedance of the upper limb under during movement. When the upper limb was in the stable state, the impedance model of the human upper limb end in the Cartesian coordinate system could be displayed as follows:

$$M\ddot{X}+B\dot{X}+KX=F$$ (5)

*M*, *B*, *K* $\in R^{3\times 3}$ respectively represented the inertial parameters, damping parameters and stiffness parameters of the human upper limb end, $X\in R^{3}$ and $F\in R^{3}$ respectively represented the position and force of the upper limb end in the Cartesian coordinate system. The position of the upper limb end was measured by the joint encoder. The joint position was calculated by the kinematic equation, and the end force was measured by the six-dimensional force sensor.

In the process of rehabilitation training, the impedance parameters of human upper limb were variable. With the changes in the rehabilitation cycle, the impedance parameters of human upper limb modified slowly. For the slow time-varying system, the recursive least square (RLS) method had its limitations. As *k* increased, the values of *P(k)* and *K(k)* decreased, resulting in declining corrections for $\overset{\wedge }{\theta_{k}}$ ,, the smaller and smaller correction effect of $\overset{\wedge }{\theta_{k}}$ from new input and output data pairs. Additionally, the accuracy of parameter estimation error decreased and the RLS method was unable to track the changes in system parameters online constantly. To overcome this shortcoming, FFRLS was carried out (Long et al., 2023).

Take the cost function:

$$J=\sum_{k=1}^{L}\lambda^{L-K}{\left[y\left(k\right)-\varphi^{T}\left(k\right)\hat{\theta }\right]}^{2}$$ (6)

λ was the forgetting factor ( 0<λ≤1 ), which meant that the input and output data were added with a time-varying weight coefficient. The weight of the latest input and output data of the *k* group was 1, and the weight coefficient of all the previous n groups was $\lambda^{n}$ . The smaller the weight coefficient of the original data was, the greater the degree of forgetting was. The values of *P(k)* and *K(k)* would not lose their ability to correct $\overset{\wedge }{\theta_{k}}$ with the increase of *k*, that is, the influence on the system parameter identification would not decrease.

The RLS derivation formula of forgetting factor was as follows:

$$\left\{\begin{array}{l} \hat{\theta }\left(k\right)=\hat{\theta }\left(k-1\right)+K\left(k\right)\left[y\left(k\right)-\varphi^{T}\left(k\right)\hat{\theta }\left(k-1\right)\right]\\ K\left(k\right)=\frac{P\left(k-1\right)\varphi \left(k\right)}{\lambda +\varphi^{T}\left(k\right)P\left(k-1\right)\varphi \left(k\right)}\\ P\left(k\right)=\frac{1}{\lambda }\left[I-K\left(k\right)\varphi^{T}\left(k\right)\right]P\left(k-1\right) \end{array}\right.$$ (7)

The method of selecting initial values $P\left(0\right),\hat{\;\theta }\left(0\right)$ was the same as RLS. The value of forgetting factor λ was generally a positive real number which was close to 1, usually greater than 0.9. In the linear system, the forgetting factor was generally 0.95≤λ≤1 . When λ=1 , the FFRLS degraded into the ordinary RLS.

#### 2.2.2 Optimal stiffness selection

At different stages of their rehabilitation, patients need different training modalities, requiring a specific stiffness from the rehabilitation robot (Zou et al., 2022). In order to increase the effectiveness of rehabilitation therapy assisted by rehabilitation robot, patients’ active participation must be encouraged by the robot controller (Luo et al., 2017; Guo et al., 2022a). At the same time, if the patient’s movement deviated from the expected movement, it should be restrained. Therefore, the reward function was set to balance patients’ participation and trajectory shift error. The reward function was defined as:

$$\begin{array}{l} r=a_{1}F_{h}V-{\left\|a_{2\;}e\right\|}^{2}\\ =\left(a_{1}F_{x}V_{x}-a_{2}^{2}e_{x}^{2}\right)+\left(a_{1}F_{y}V_{y}-a_{2}^{2}e_{y}^{2}\right) \end{array}$$ (8)

$F_{h}V$ was the output power of the patient, which was used to measure the effort of the patient; $e_{x}$ and $e_{y}$ were the trajectory error at the end of Cartesian space; $a_{1}$ and $a_{2}$ were the parameters which struck a balance between the patient’s effort and the trajectory deviation. When the reward value was higher, the higher the patient’s participation in rehabilitation training was higher and the deviation of the expected trajectory was less.

$$\begin{array}{l} F_{x}=K_{hx}\left(x_{d}-x\right)-B_{hx}V_{x}\\ F_{y}=K_{hy}\left(y_{d}-y\right)-B_{hy}V_{y} \end{array}$$ (9)

Eq. 9 was substituted into Eq. 8,

$$r=\left(K_{hx}e_{x}V_{x}-a_{1}B_{hx}V_{x}^{2}-a_{2}^{2}e_{x}^{2}\right)+\left(K_{hy}e_{y}V_{y}-a_{1}B_{hy}V_{y}^{2}-a_{2}^{2}e_{y}^{2}\right)$$ (10)

The reward function *r* took the partial derivative with respect to $e_{x}$ and $e_{y}$ respectively.

$$\begin{array}{l} \frac{\delta r}{\delta e_{x}}=a_{1}K_{hx}V_{x}-2a_{2}^{2}e_{x}\\ \frac{\delta r}{\delta e_{y}}=a_{1}K_{hy}V_{y}-2a_{2}^{2}e_{y} \end{array}$$ (11)

In order to maximize the reward function, $\frac{\delta r}{\delta e_{x}}=0,\frac{\delta r}{\delta e_{y}}=0$ ,

$$\begin{array}{l} {\hat{e}}_{x}=\frac{a_{1}K_{hx}V_{x}}{2a_{2}^{2}}\\ {\hat{e}}_{y}=\frac{a_{1}K_{hy}V_{y}}{2a_{2}^{2}} \end{array}$$ (12)

During rehabilitation training, the inertia, motion acceleration, and speed of the rehabilitation robot were very small. The inertia force and Coriolis force could be safely disregarded. In addition, compared with the joint torque of the rehabilitation robot, friction was also found to be negligible. Assuming that the affected limb end achieved a stable state within a short time, the force of the rehabilitation robot was equal to that exerted by the patient:

$$\begin{array}{l} F_{rx}+F_{x}=0\\ F_{ry}+F_{y}=0 \end{array}$$ (13)

$$\begin{array}{l} \left(K_{rx}+K_{hx}\right)e_{x}-\left(B_{rx}+B_{hx}\right)V_{x}=0\\ \left(K_{ry}+K_{hy}\right)e_{y}-\left(B_{ry}+B_{hy}\right)V_{y}=0 \end{array}$$ (14)

Eq. 12 was substituted into Eq. 14 to obtain the optimal stiffness of impedance control of rehabilitation robot:

$$\begin{array}{l} {\hat{K}}_{rx}=\frac{2a_{2}^{2}\left(B_{rx}+B_{hx}\right)}{a_{1}K_{hx}}-K_{hx}\\ {\hat{K}}_{ry}=\frac{2a_{2}^{2}\left(B_{ry}+B_{hy}\right)}{a_{1}K_{hy}}-K_{hy} \end{array}$$ (15)

${\overset{\wedge }{K}}_{\text{rx}}$ and ${\overset{\wedge }{K}}_{\text{ry}}$ were the optimal stiffness of the impedance control of the rehabilitation robot, which maximized the reward function during rehabilitation training of the affected limb. As demonstrated by Eq. 15, the optimal stiffness of the robot’s impedance control was inversely proportional to the stiffness of the affected limb, which was conducive to providing corresponding feedback and parameter changes according to the different needs and actual state of patients during rehabilitation training. When the capacity of the affected limb decreased, the assisting force of the rehabilitation robot increased. The larger the value of the parameter $a_{1}$ was, the smaller the optimal stiffness value of the rehabilitation robot was. In other words, more attention should be paid to the effort of the affected limb during rehabilitation training to satisfy the definition of the reward function. The size of the stiffness parameter was definitely associated with the level of assistance of the rehabilitation robot (Honghai et al., 2022).

When the affected limb had minimal participation ( $K_{hx}\approx 0,K_{hy}\approx 0$ ), the stiffness of the rehabilitation robot tended to be infinity. The following limits were set for the stiffness of the impedance control to avoid this situation. $K_{\min}$ and $K_{\max}$ were the minimum and maximum stiffness that the rehabilitation robot controller could provide.

$$K_{r}=\max\left\{K_{\min},\min\left\{K_{\max},{\hat{K}}_{r}\right\}\right\}$$ (16)

## 3 Experiment results

In order to verify the impedance identification algorithm and the adaptive impedance control technology proposed in this study, three sets of experiments were carried out in this section: impedance parameter identification verification and variable impedance control simulation experiment, as well as the adaptive impedance control verification.

### 3.1 Impedance parameter identification verification

**FIGURE 5:**
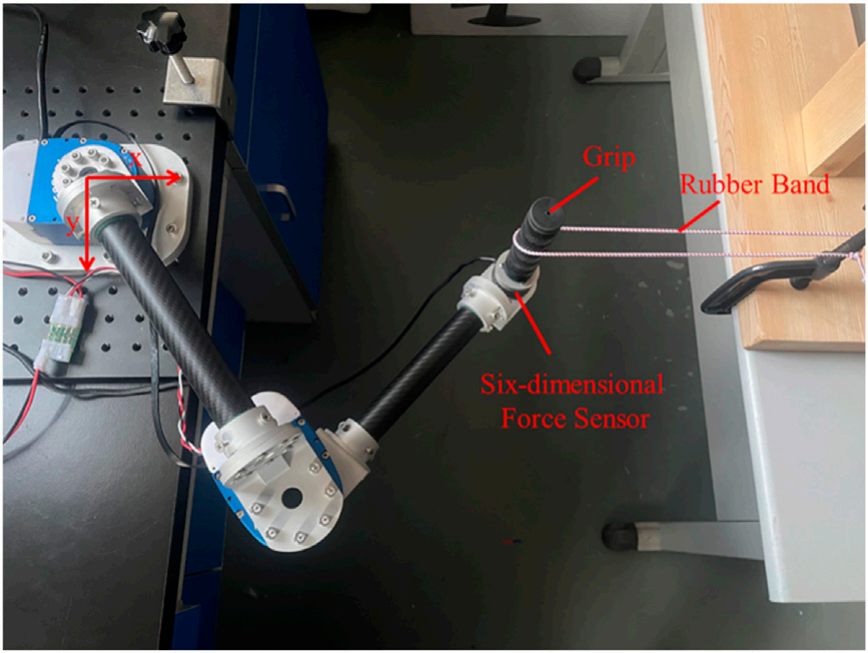
The impedance identification experiment platform.

As shown in Figure 5, the platform for impedance identification experiment was set up. The end handle of the rehabilitation robot was connected to the elastic body (rubber band). The other end of the elastic body was fixed, and the elastic body was fixed stiffness within a certain range. The six-dimensional force sensor with the end connected to the grip could measure the force and the torque in three directions in Cartesian space. The Cartesian coordinate system was installed at the rotation center of the first joint. Since the experiment platform belonged to the tabletop upper limb rehabilitation robot, only coordinate systems in the *x* and *y* directions were established.

**FIGURE 6:**
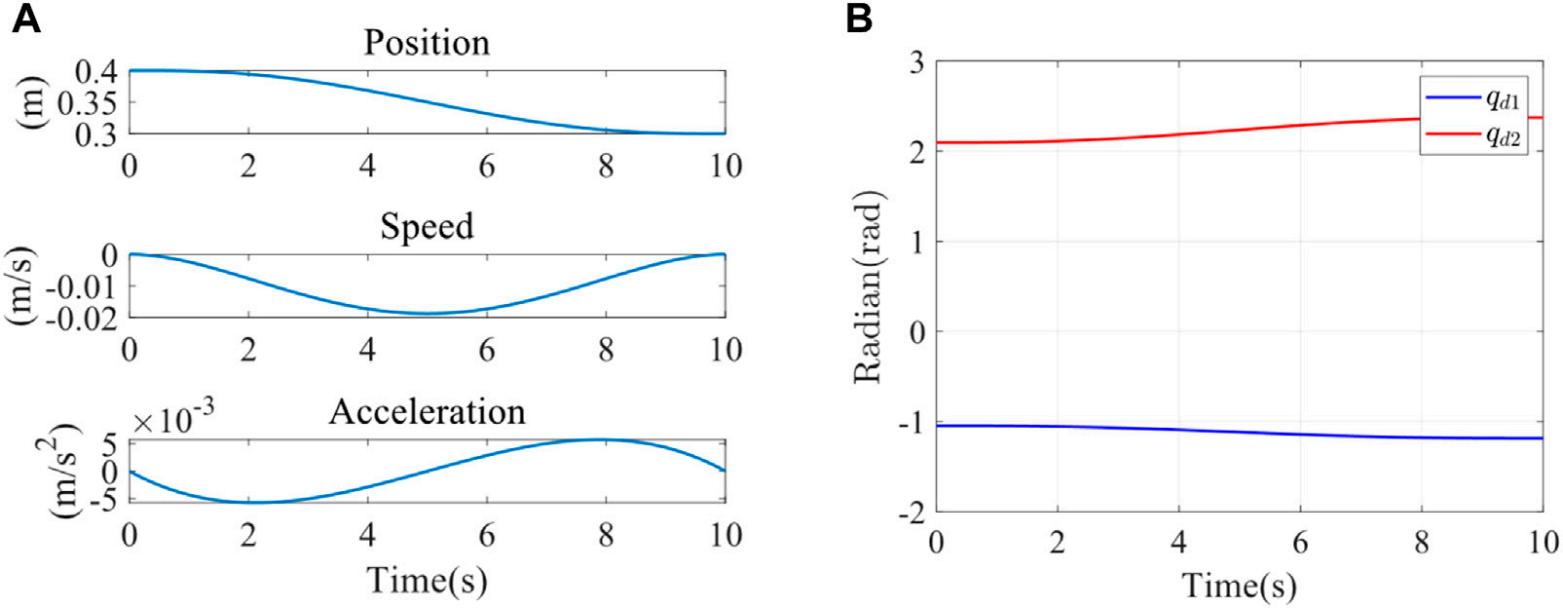
**(A)** *X*-direction trajectory planning in Cartesian space. **(B)** Joint 1 and joint 2 expected trajectory.

Since the peak moment of the joint was 19 Nm, a spring with excessive stiffness could not be used for the impedance parameter identification experiment. Therefore, a rubber band chosen for the experiment had an elastic stiffness of 25 N/m. The end stiffness parameters were varied by changing the number of strands, stiffness, and position of the rubber bands. Firstly, two strands of rubber bands were selected for the impedance parameter identification experiment. The initial point of the end was (0.4 m, 0), and the movement was planned to (0.3 m, 0). The trajectory planning adopted the quintic polynomial interpolation method. Under the initial condition of the experiment, the elastic band was just taut, and the force sensor could detect the tension of the elastomer at the end, which was in the same plane as the elastomer at the other fixed end. It was planned to move from point A (0.4 m, 0) to point B (0.3 m, 0). The trajectory planning results in the x direction were shown in the following figure using quintic polynomials. The position, speed, and acceleration of the end from top to bottom were illustrated in Figure 6A. It could be observed that the speed and acceleration in the initial and terminal states were 0. This method could successfully avoid the impact of the rehabilitation robot on the motor during the process of starting and stopping. Meanwhile, the smooth trajectory also made the rehabilitation process more steady, which was beneficial to the rehabilitation of the affected limb. The expected trajectories of the two joints were obtained by inverse kinematics, as shown in Figure 6B, $q_{d1}$ and $q_{d2}$ were input to the joint servo controller of the robot as the position control of the two joints controller.

**FIGURE 7:**
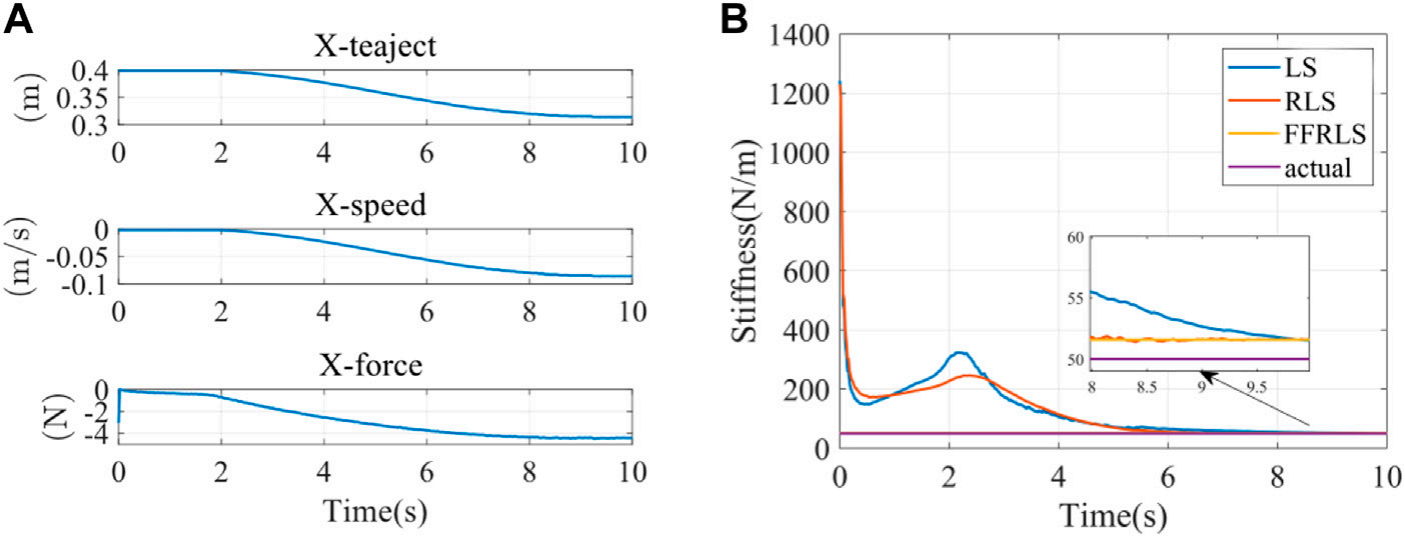
**(A)** The input parameters of impedance identification experiment **(B)** Impedance parameter identification results.

The interaction force F between the end of the elastomer and the rehabilitation robot was detected by the force sensor. The real-time joint angle *q* was obtained by the encoder of the rehabilitation robot. The real-time angles of two joints *q* acquired terminal position through the forward kinematics. The terminal speed was obtained by the differential. Inputting the terminal position, terminal speed, and terminal interaction force, the terminal impedance parameters are estimated by the least square method (LS), RLS, and FFRLS. The input parameters of the impedance identification experiment were displayed in Figure 7A. The terminal impedance parameters estimated by LS, RLS, and FFRLS were shown in Figure 7B The blue, red, and yellow lines represented the estimated end stiffness of the LS, RLS, and FFRLS, respectively, while the purple line represented the actual stiffness value. It illustrated that RLS and LS began to converge after 3s, much slower than FFRLS.

Impedance parameter identification errors were shown in Table 2. Since the stiffness estimation of the first few seconds by RLS and LS was divergent, it did not have statistical significance. All data in Table 2 were calculated after the stiffness identification curves of FFRLS, RLS, and LS. The root-mean-square errors of stiffness identification by FFRLS ( λ = 0.95), RLS, and LS were 1.5900 N/m, 1.6075 N/m and 2.0703 N/m, respectively. The maximum stiffness identification errors were 1.5900 N/m, 1.6859 N/m, and 2.6888 N/m, respectively. The results showed that the root-mean-square error and maximum error of the FFRLS ( λ = 0.95) stiffness estimation were smaller than those of RLS and LS. Therefore, the stiffness estimation from FFRLS had the best result.

**TABLE 2:** Impedance parameter identification errors.

Identification stiffness	RMS(N/m)	MAX (N/m)
FFRLS ( λ = 0.95)	1.5900	1.5900
RLS	1.6075	1.6859
LS	2.0703	2.6888

### 3.2 Variable impedance control simulation verification

The feasibility of the above impedance control was verified by simulation in Matlab 2023a. To verify the system’s ability of control stiffness under the external disturbances, we simulated the stiffness of the upper limb end of the healthy participants by modifying impedance parameters, thereby altering the system’s stiffness behavior. This demonstrated its control capability over impedance characteristics. The simulation platform was set up based on actual platform parameters. The parameters of kinematic model were set as follows*: m*_*1*_ = 1 kg, *m*_*2*_ = 0.7 kg, *I*_*1*_ = 0.25, *I*_*2*_ = 0.1, *l*_*l*_ = *l*_*2*_ = 0.4, *l*_*c1*_ = *l*_*c2*_ = 0.2; *m*_*1*_ and *m*_*2*_ were the masses of rods A and B respectively. *l*_*l*_ and *l*_*2*_ were the lengths of rods A and B respectively. *l*_*c1*_ and *l*_*c2*_ were the distances from the center of mass of rods A and B to the rotation center, respectively. *I*_*1*_ and *I*_*2*_ were the moments of inertia of rods A and B, respectively. Parameter *g* represented the gravitational acceleration and was taken as 9.8 m/s. The control stiffness parameter was established as follows:

$$K_{d}\left(t\right)=diag\left\{10+10\sin\left(2t\right),10+10\cos\left(2t\right)\right\}$$ (17)

The end load of Cartesian coordinate system was established as follows:

$$f_{e1}=2\sin\left(2t\right),f_{e2}=2\cos\left(2t\right)$$ (18)

**FIGURE 8:**
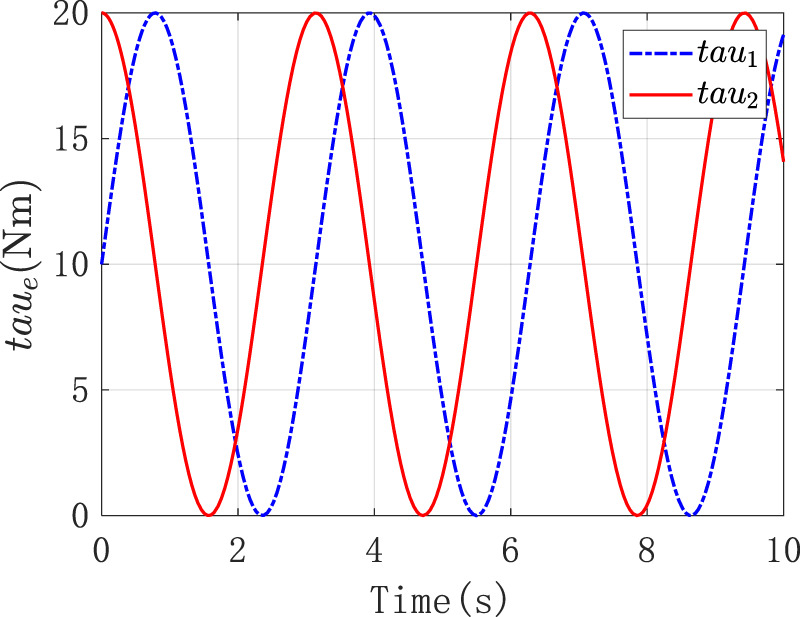
The stiffness of two different joints of the robot.

That is, the stiffness changed at a fixed frequency within a certain range, which was reflected in the varying stiffness of the manipulator’s end in different directions on the plane. As shown in Figure 8, the solid and dashed lines were the curves of the stiffness of the two different joints of the robot over time.

**FIGURE 9:**
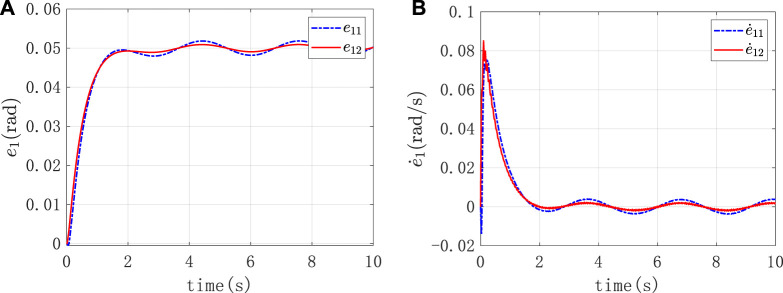
**(A)** The position tracking error caused by the impedance control **(B)** The derivative change curve caused by the impedance control.

Under the above external conditions, the corresponding load force was applied to it. And it was expected that the resulting torque output and error performance could reflect the stiffness control performance. Figures 9A,B were the position tracking error and the derivative change curve caused by the impedance control of the two joints of the robot, respectively. As observed in Figure 9A, in the face of the load imposed by the external environment, the tracking error $e_{1}$ of the reference position converged in a small neighborhood where the equilibrium point was 0 and the steady-state error did not exceed 0.06. This result indicated the effectiveness of the adaptive impedance control strategy when the platform faced the variable impedance. As shown in Figure 9B, the first derivative of the reference position tracking error ${\dot{e}}_{1}$ gradually converged to 0, which indicated that the position error of the platform gradually stabilized under the variable load force.

**FIGURE 10:**
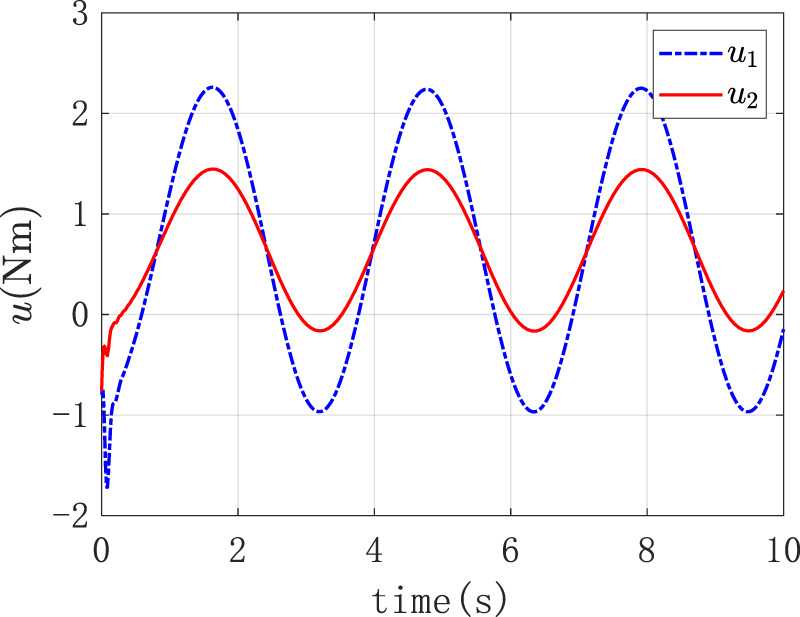
The output torque of the two joints.

Figure 10 was the graph of the output torque of the two joints changing over time, and it displayed that the joint itself output the corresponding output torque to counteract the external input torque.

### 3.3 Adaptive impedance control verification

**FIGURE 11:**
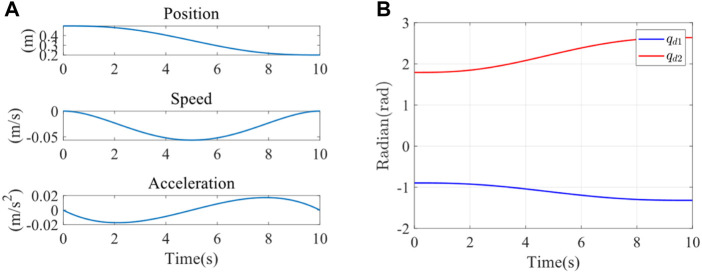
**(A)** *X*-direction trajectory planning **(B)** Expected trajectory of joint 1 and joint 2.

To verify the adaptive impedance control system in this study, a healthy male participant (24 years old, 1.88 m in height, 84 kg in weight) was recruited in the experiments, as shown in Figure 1, The study was reviewed by Shanghai University of Medicine and Health Sciences ethics, batch number 2022-ZYXM4-04-420300197109053525. The experiment was designed as follows: the rehabilitation task required the subject to move the end of the upper limb from A (0.5 m, 0) to C (0.2 m, 0), and each training time was 10s. Under the condition of constant impedance control and adaptive impedance control, the experiments were carried out with varying participation of the affected limb (i.e., different impedance parameters). The trajectory planning results of x direction using quintic polynomials were reported in Figure 11A, including the position, speed, and acceleration of the end from top to bottom. At the starting point A and the end point C, there was no speed or acceleration. This approach effectively reduced the impact of the rehabilitation robot on the motor during the phases of starting and stopping. Furthermore, the well-executed trajectory enhanced the overall stability of the rehabilitation process, thereby promoting the recovery of the affected limb. The expected trajectories of both joints were determined by inverse kinematics, as illustrated in Figure 11B. $q_{d1}$ and $q_{d2}$ were input to the joint servo controller of the rehabilitation robot as the position control of the two joints controller.

**FIGURE 12:**
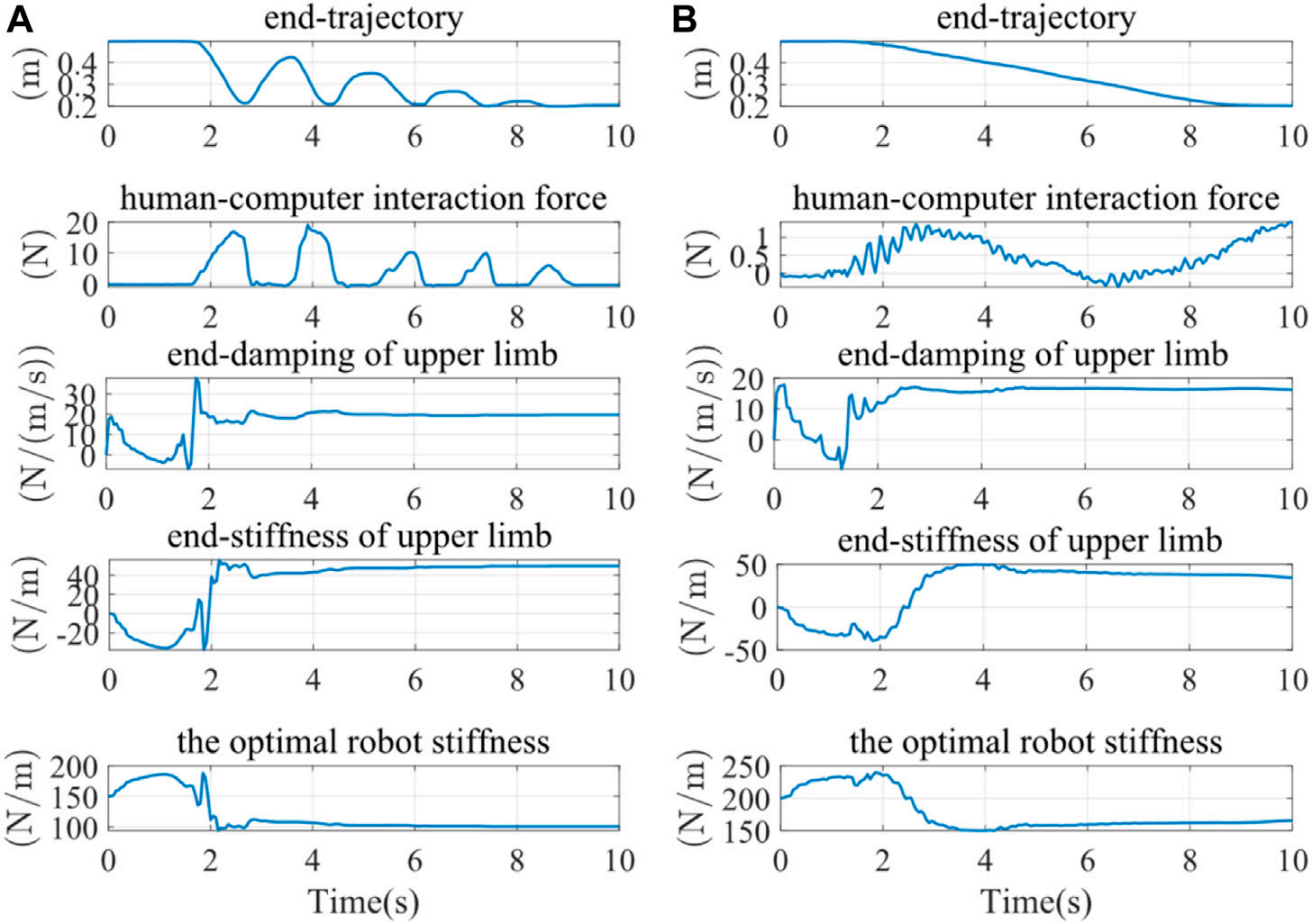
The results of experiments under different conditions of the participation. **(A)** High participation experiment results **(B)** Low participation experiment results.

The parameters $a_{1}=10,a_{2}=2$ were set to make the weight of the work performed by the human upper limb higher in the rehabilitation strategy. The upper limit of the optimal stiffness was $K_{\max}$ = 400 N/m, while the lower limit was $K_{\min}$ = 10 N/m. The impedance limit could protect the affected limb and improve the safety of the rehabilitation training better. The experiment results under different participation conditions were illustrated in Figure 12, including the end-trajectory, human-computer interaction force, the identified end-damping, end-stiffness of the upper limb, and the robot’s optimal stiffness. Figures 12A,B was the result of the subject’s high and low participation. When there is a high level of the subject’s participation in upper limb rehabilitation training, the stiffness at the upper limb’s end is high, and the optimal robot stiffness is low, indicating a lower degree of robot assistance. As a result, a larger degree of robot assistance was indicated when there was a low participation level in upper limb rehabilitation training, low stiffness at the upper limb’s end, and high optimal robot stiffness.

**FIGURE 13:**
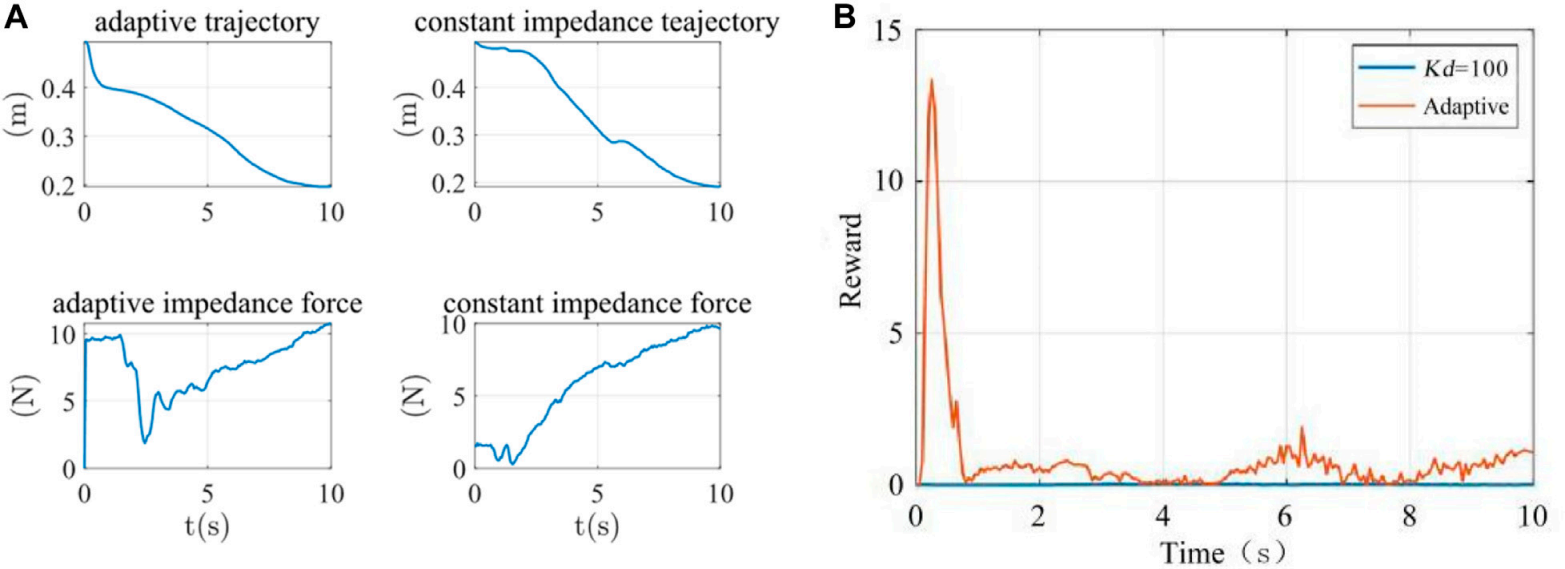
**(A)** The terminal trajectory and interaction force **(B)** Reward function.

The terminal trajectories and interaction forces for both the constant impedance control (*Kd* = 100 N/m) and the adaptive impedance control were shown in Figure 13A. In both experiments, the interactive forces of adaptive impedance and constant impedance consistently showed high participation levels for the affected limb. Demonstrating that The reward obtained from the adaptive impedance control during the rehabilitation training was significantly higher than that of the constant impedance control with *Kd* = 100 N/m, as illustrated by the reward functionsin Figure 13B. This confirmed the effectiveness and robustness of the adaptive impedance control strategy proposed in this study.

The analysis of the reward function was shown in Table 3. The average rewards of constant impedance control (*Kd* = 100 N/m) and adaptive impedance control were 0.0152 and 0.8514, and the maximum rewards were 0.0471 and 13.3437, respectively.

**TABLE 3:** The analysis of reward function.

Control strategy	The average reward	The maximum reward
Constant impedance control (*K*_*d*_ = 100 N/m)	0.0152	0.0471
Adaptive impedance control	0.8514	13.3437

## 4 Discussion

In this study, we constructed a mechanical platform and developed a novel adaptive impedance control strategy for the upper limb rehabilitation robot. We utilized a mass-spring-damping system to simulate the musculoskeletal system. With the changes in rehabilitation cycle, we used FFRLS to improve the accuracy of parameter estimation error. This method, in contrast to earlier LS or RLS, could constantly track changes in the impedance parameters online and did not decrease system parameter identification due to increased stiffness. We employed the reward function to strike a balance between the subject’s participation and the trajectory deviation error, further achieving the optimal stiffness of impedance control of the rehabilitation robot.

Various techniques were employed in some studies to estimate and adjust participants’ optimal stiffness. An algorithm that could adaptively change the impedance control’s stiffness parameters in response to the observed values of the interaction force between patients and robots was proposed by Riener *et al.* Through the linear adaptive law, when the workload of the patient was detected to increase, the stiffness value was reduced (Riener et al., 2005). Ground on the evaluation of human active torque, Shahid *et al.* employed a similar method to control the stiffness of the manipulator (Hussain et al., 2013). Although their methods achieved control results, this study fully considered the levels of the subject’s participation and enthusiasm in rehabilitation training in the form of a reward function. Patients’ active participation awareness played a significant role in promoting the effect of rehabilitation training (Pawlak et al., 2022).

Moreover, this study designed the experiments under different participation to get the different parameters from the robot. When patients showed the signs of fatigue or reduced movement ability, the robot could increase the assistance level to maintain training continuity and efficacy, avoiding potential secondary injuries or training outcomes (Yang et al., 2023). Conversely, when patients exhibited a high level of participation, the robot might reduce its assistance to encourage patients to make more use of their own muscle, which supported neural plasticity and the rehabilitation of motor functions (Kawahira et al., 2010). Further studies via this approach enables more personalized rehabilitation training, satisfying the specific needs of different patients, thereby improving the efficiency of rehabilitation and accelerating the patient’s return to normal life and work.

In the simulation experiment, it was observed that the corresponding torque output at the end of the robotic arm could resist the corresponding load force when the platform was facing variable external load force and the error was controlled within a narrow range, proving the effectiveness of the adaptive impedance control strategy. The limit of the simulation was that the stiffness change law was set by ourselves to simulate the actual situation. However, the output stiffness value of the assist-as-needed strategy was optimized according to the stiffness of the affected limb. We will optimize the experimental settings by taking assist-as-needed rehabilitation procedures into account in subsequent studies.

Since impedance control achieved regulation and stabilization of robot motion by establishing a mathematical relationship between the interaction forces and the reference trajectories (Al-Shuka et al., 2018), we compared adaptive impedance control and constant impedance control for experimental verification. By setting different parameters to simulate varying levels of participant engagement, the results obtained were consistent with the experiment in which a healthy subject was involved. We also obtained that the average and maximum rewards of adaptive impedance control were higher than those of constant impedance control at *K*_*d*_ = 100 N/m. Luo, Duan, and Berenice conducted comparative simulation experiments on constant impedance control and variable impedance control (Luo et al., 2017; Maldonado et al., 2015; Duan et al., 2018). In these researches, Luo used different levels of simulated stiffness values, Duan compared the two methods in different environments, and Berenice simulated the situations of subjects under different task modes. Their research findings indicated that adaptive impedance control had better force tracking performance and potential for facilitating rewards compared to constant impedance control. Adaptive impedance control technology can be utilized in robot-assisted rehabilitation systems under various conditions which further prove the effectiveness of adaptive impedance control in rehabilitation training. Ibarra and Wang also suggested adaptive impedance control strategies, considering the influence of patients on the ankle rehabilitation robot and adjusting the robot aids in real time (Perez-Ibarra et al., 2015; Wang et al., 2019). The intervention of the exoskeleton was considered in the process of training (Guo et al., 2022b).

This control strategy offered significant potential for achieving the best active training effect and creating a controllable impedance environment for the patient. The adaptive control strategy can improve the performance of the human-robot interaction and the effectiveness of the control system for upper limb rehabilitation robot. In addition, the proposed strategy could also be applied to the different rehabilitation robots. In our follow-up studies, we will test the proposed method with more healthy subjects and patients to accurately identify the differences based on the different participation, and we will also apply this control system for the wearing assistive devices to test its effectiveness, improving the rehabilitation efficacy eventually.

## 5 Conclusion

In this study, an novel adaptive impedance strategy for upper-limb rehabilitation robots was proposed. The efficacy of optimal stiffness control was confirmed through a comparison of performance across various levels of upper limb participation during the rehabilitation process. A comparison of rehabilitation performance between adaptive impedance control and consant impedance control was also conducted. The simulation and the experiments fully verified the effectiveness of this adaptive impedance control strategy.

## Data Availability

The original contributions presented in the study are included in the article/Supplementary Material, further inquiries can be directed to the corresponding author.
