# Supplementary figures and images for: Research on adaptive impedance control technology of upper limb rehabilitation robot based on impedance parameter prediction
Source: Front Bioeng Biotechnol. 2024 Jan 3;11:1332689. doi: 10.3389/fbioe.2023.1332689 (PMC10792012; doi:10.3389/fbioe.2023.1332689)

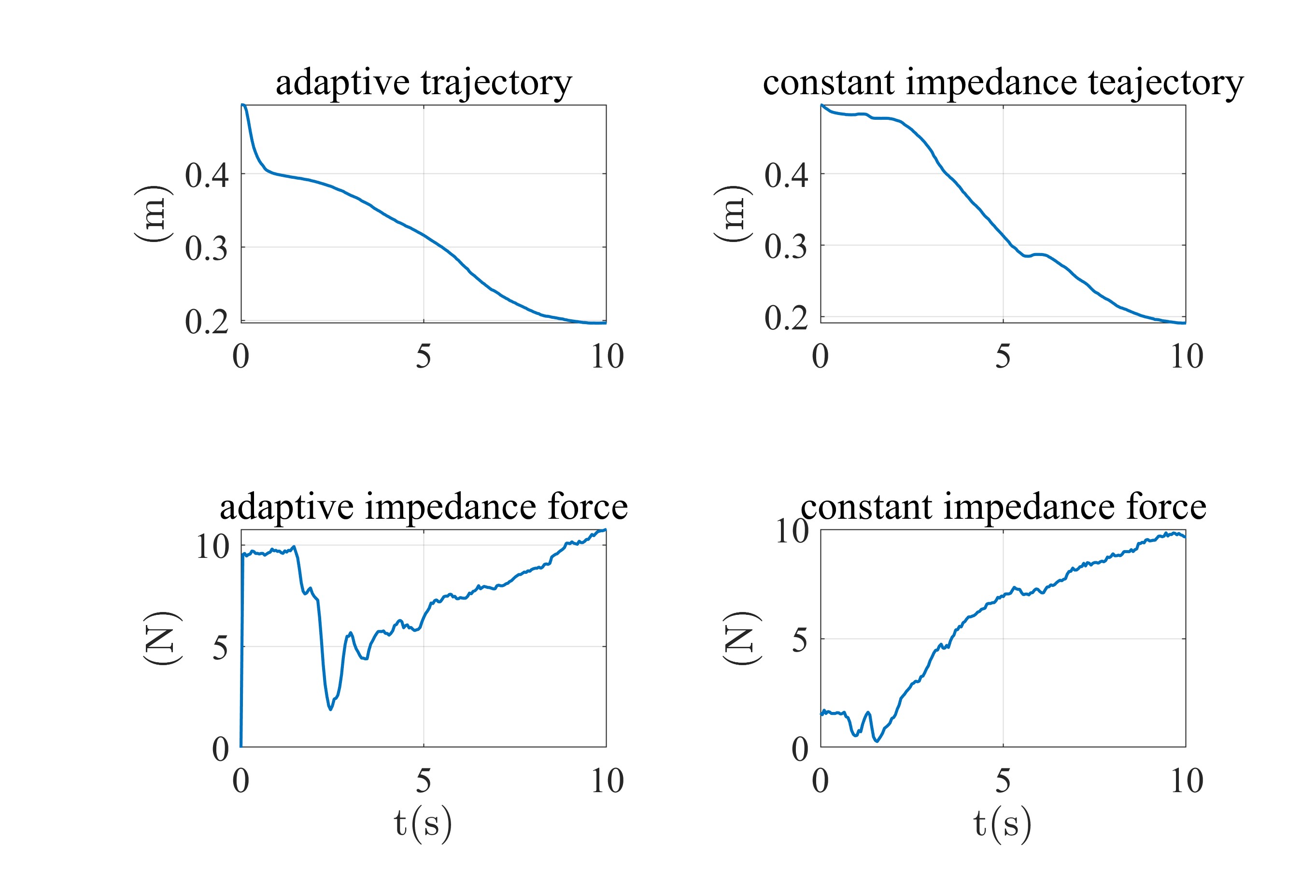

Supplement: Supplementary file 1 [file Image3.JPEG]

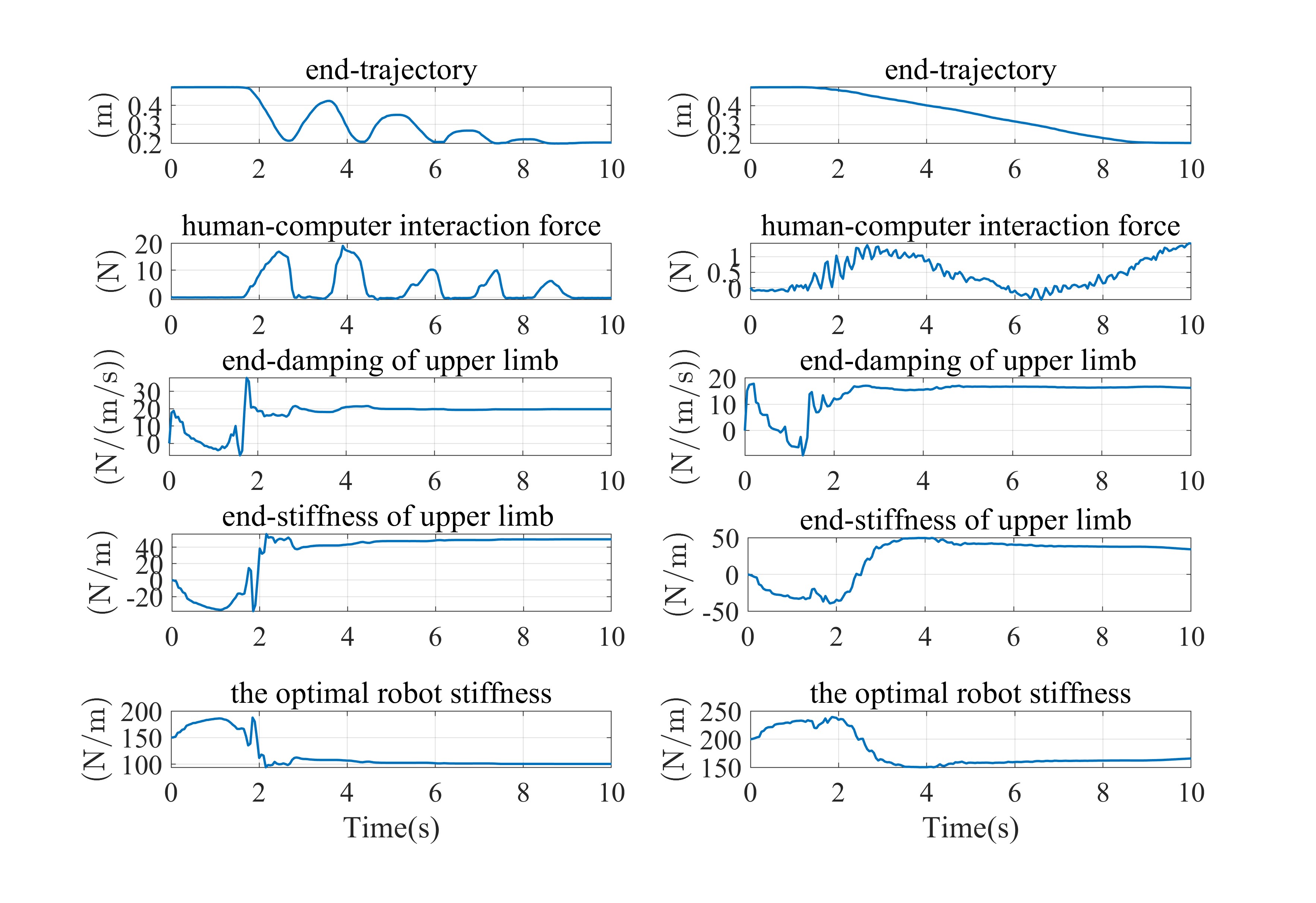

Supplement: Supplementary file 2 [file Image1.JPEG]

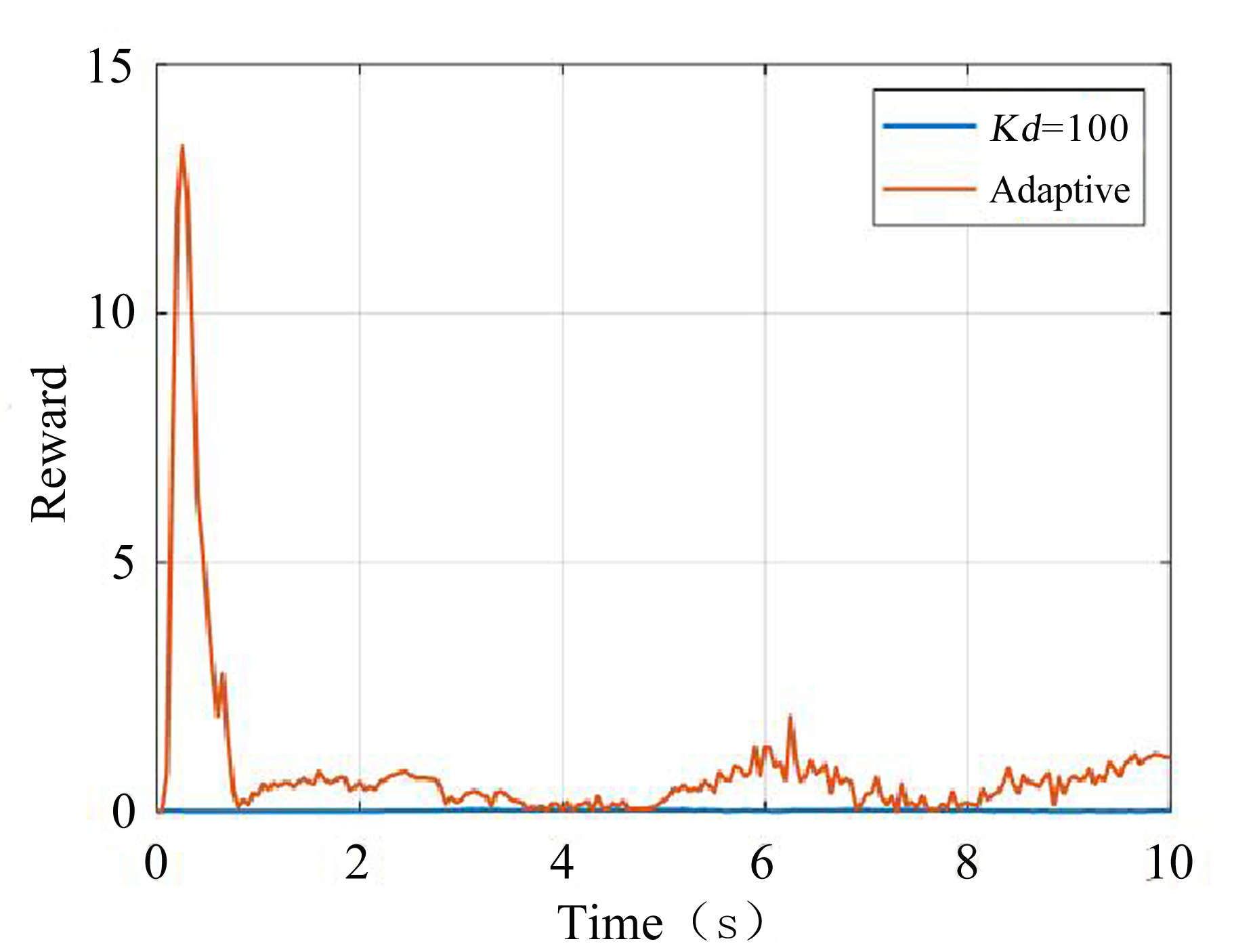

Supplement: Supplementary file 3 [file Image2.JPEG]
